# Initiative for standardization of the format of the next-generation sequencing (NGS) results

**DOI:** 10.15190/d.2015.36

**Published:** 2015-05-19

**Authors:** Veronika Pipan, Tanja Kunej

**Affiliations:** Department of Animal Science, Biotechnical Faculty, University of Ljubljana, Slovenia

**Keywords:** Cancer, database, next generation sequencing (NGS), single nucleotide polymorphism (SNP)

## Abstract

The number of published reports using next-generation sequencing (NGS) technology in cancer research is increasing. These technologies generate large amounts of data that need to be appropriately presented and available to other researchers for further use. Our goal was to create a comprehensive database with single nucleotide polymorphisms (SNPs) associated with different types of cancer to integrate them to our bioinformatics tools. We reviewed more than 200 scientific papers and extracted relevant information on mutations detected by NGS technology. The current version of the database contains more than 100.000 mutations in more than 70 types of cancer. However, our review of NGS studies revealed great variation in presentation of NGS data in scientific literature with almost no effort for standardization of the data format. NGS results are published in a variety of forms which hinders the gathering of information. Therefore we suggested a uniform format for presenting the NGS data. This will allow faster database development, easier access and data sharing between the laboratories. The database will be a useful tool to many researchers in the field of cancer research and can be a base for a range of studies such as genome-wide association studies, microRNA target binding, and development of cancer biomarkers research.

Next-generation sequencing (NGS), also known as massively parallel sequencing, is rapidly transforming biomedical and biological research from single gene to genome scale^1-3^. Within only a few years of the advent of NGS technologies, it is now possible to allow researchers to apply whole-exome sequencing (Exome-Seq), whole-genome sequencing (WGS), whole-transcriptome sequencing (RNA-Seq), or a combination of them to investigate individual genomes, especially those related to disease. Next-generation sequencing technologies have demonstrated their power in detecting disease causing or causative genetic variants of human diseases^2,4,5^, especially in cancer^2,6,7^. The use of NGS technology in cancer studies and the number of publications is rapidly increasing. Consequently, a massive amount of data is generated. There are already databases available online with an attempt to summarize this data and present it in a comprehensive way. Next Generation Sequencing Catalog (NGS Catalog, http://bioinfo.mc.vanderbilt.edu/NGS/)^2^, collects scientific literature on NGS studies with hyperlinks to the publications. International Cancer Genome Consortium Data Portal (ICGC, https:// dcc.icgc.org/) provides tools for visualizing, querying and downloading the data released by the consortium's member projects. They systematically and comprehensively characterize somatic mutations in 50 different cancer types and subtypes using high-throughput NGS technologies. Their goal is to rapidly bring these data to the cancer research community in order to accelerate studies on the discovery of cancer causes, to enhance the accuracy of diagnosis and to improve treatments. Catalogue Of Somatic Mutations In Cancer (COSMIC, http://cancer.sanger.ac.uk/cosmic)^8^ is designed to store and display somatic mutation information and related details and contains information relating to human cancers.

As the number of published articles with various findings and results of NGS technology in cancer is growing, it is important that the data is collected and presented in a suitable way. Our goal was to create an organized and comprehensive database with mutations in different types of cancer, that will be integrated into our previously developed bioinformatics tools, such as the miRNA SNiPer. This enables identification of polymorphisms residing within miRNA genes^9^. We reviewed the scientific literature and databases on NGS studies in cancer research and collected relevant information on mutations detected by NGS technology. Furthermore, our literature review revealed that scientific papers of different research groups vary greatly and researchers publish their findings in a variety of forms. This makes gathering information from them quite a challenge; therefore a uniform format for presenting the results is needed.

We collected over 200 papers from databases: PubMed, PubMed Central, ScienceDirect, and NGS Catalog using keywords such as »*cancer*«, »*mutations*«, »*whole genome sequencing*«, »*exome sequencing*«, and »*next generation sequencing*«. We gathered relevant information on mutations detected by NGS technology: gene name (approved by HUGO Gene Nomenclature Committee), chromosome number, Ensembl gene ID, Entrez gene ID, transcript information, rs number (if available), position of the mutation in the genome, cDNA and protein (amino acid replacement with single letter code), type of cancer, mutation variant, technology used (platform), digital object identifier (DOI), reference and hyperlink to the publication. The data were collected in an Excel table. Based on the systematic review of over 200 papers describing the NGS in cancer research, we proposed a simple, intelligible, easily accessible and ready-to-use database containing fifteen relevant pieces of information on each mutation and hyperlink to the publication.

The use of NGS technology in cancer research is increasing and as a result a growing amount of data is generated. With this in mind we created a comprehensive database of 109.028 mutations associated with more than 70 different types of cancer. There are almost 12.000 SNPs with known reference SNP ID number (rs number) in our database. Those will be integrated into the miRNA SNiPer tool for subsequent functional annotation of miRNA genes. According to our extensive literature review we also developed a template for standardization of NGS data presentation. Each column of the database represents a category of information (e.g., gene name, type of cancer, etc.) and each row represents a single mutation in a particular gene (**Figure 1**). The database includes diverse types of information on mutations and is designed in order to make the data easily accessible for further use.

**Figure 1:**
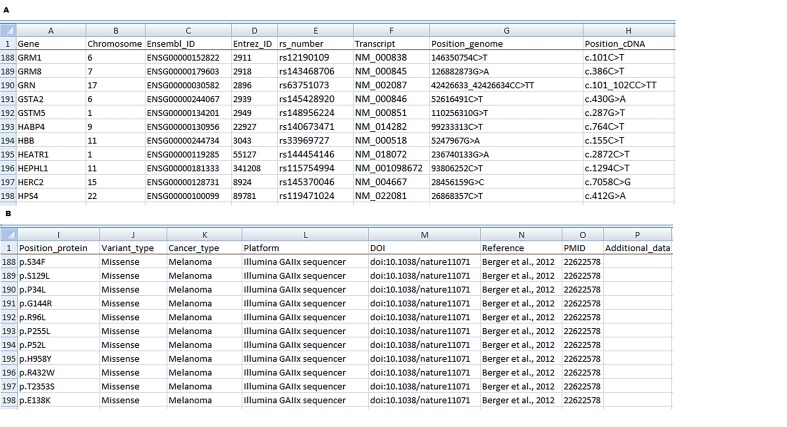
Suggested format for the presentation of NGS results ****A)**** Column A: Gene name, B: Chromosome number, C: Ensembl ID, D: Entrez ID, E: Reference SNP ID number, F: Transcript accession number, G: Position of mutation in the genome, H: Position of mutation in cDNA; ****B)**** I: Position of mutation in protein, J: Variant type; K: Cancer type, L: Sequencing technology (platform), M: Digital object identifier (DOI), N: Reference, O: PubMed unique identifier (PMID), P: Additional data

Our literature review revealed that most of the research groups that perform NGS studies do not focus much on data standardization, which is an important aspect for the data being available to other researchers. Scientific papers of different research groups vary greatly and researchers publish their findings in a variety of forms, which makes gathering information from them challenging. For example, some publications have attached Excel files with transparently sorted data^10,11^ which makes final data editing easier. However, in some cases the data is stored in a .txt, .doc or .pdf formats, or tables rotated by 90°. Data presented like this is difficult to manage and each piece of information has to be extracted manually, which is very time-consuming. Some publications are very well written with easily accessible results, while others hinder further use of the results.

NGS data are extensive; therefore a uniform and simple format for presenting the results is needed. Our database could represent a template and an example for other researchers in order to make the increasing amount of data more transparent and easily available. Standardization of the format for NGS data presentation will facilitate further development of our database as well as help other researchers in NGS and cancer studies. The data from the database can be sorted according to different types of information in order to create specialized databases, for example, based on preferred individual cancer types. It can also be analyzed using various bioinformatics tools. It can be a useful tool in a search of new gene hubs and potential biopathways. Our database will be useful to researchers involved in genome-wide association studies, development of cancer biomarkers, prioritization of genomic loci, for further functional studies or genomic overlap analysis, like overlap with QTL, miRNA genes and miRNA binding sites.
